# Motion of Adsorbed Nano-Particles on Azobenzene Containing Polymer Films

**DOI:** 10.3390/molecules21121663

**Published:** 2016-12-03

**Authors:** Sarah Loebner, Joachim Jelken, Nataraja Sekhar Yadavalli, Elena Sava, Nicolae Hurduc, Svetlana Santer

**Affiliations:** 1 Department of Experimental Physics, Institute of Physics and Astronomy, University of Potsdam, 14476 Potsdam, Germany; saphery@web.de (S.L.); jelken@uni-potsdam.de (J.J.); 2 Nanostructured Materials Laboratory, The University of Georgia, Athens, GA 30602, USA; ynsekhar@outlook.com; 3 Gheorghe Asachi Technical University of Iasi, Department of Natural and Synthetic Polymers, Prof. Dimitrie Mangeron Street, 73, 700050 Iasi, Romania; elenasavaa@gmail.com (E.S.); nhurduc@ch.tuiasi.ro (N.H.)

**Keywords:** motion of adsorbed nano-particles, azobenzene containing polymer films, fluctuating surfaces

## Abstract

We demonstrate in situ recorded motion of nano-objects adsorbed on a photosensitive polymer film. The motion is induced by a mass transport of the underlying photoresponsive polymer material occurring during irradiation with interference pattern. The polymer film contains azobenzene molecules that undergo reversible photoisomerization reaction from *trans*- to *cis*-conformation. Through a multi-scale chain of physico-chemical processes, this finally results in the macro-deformations of the film due to the changing elastic properties of polymer. The topographical deformation of the polymer surface is sensitive to a local distribution of the electrical field vector that allows for the generation of dynamic changes in the surface topography during irradiation with different light interference patterns. Polymer film deformation together with the motion of the adsorbed nano-particles are recorded using a homemade set-up combining an optical part for the generation of interference patterns and an atomic force microscope for acquiring the surface deformation. The particles undergo either translational or rotational motion. The direction of particle motion is towards the topography minima and opposite to the mass transport within the polymer film. The ability to relocate particles by photo-induced dynamic topography fluctuation offers a way for a non-contact simultaneous manipulation of a large number of adsorbed particles just in air at ambient conditions.

## 1. Introduction

The very active research on manipulation of small objects at nano- and micro-scale emerges out of the growing importance of nanotechnology as a key research field offering new and revolutionary solutions for building sensors, developing strategies for self-repairing complex and integrated structures, the creation of new materials such as quantum aggregates, or hybrid bio-electro-mechanical systems, among others [1]. In these research fields, the physical problem in spatial and temporal control of the adsorbed micro/nano-objects is that the objects are being trapped at the surface due to strong adhesion forces. In fact, the surface forces on small particles are so strong that one cannot use any existing approaches for manipulating tiny objects such as optical tweezers [2], optically induced dielectrophoresis [3,4], NEMS, and many more [5]. These techniques generate forces on small particles of the order of hundreds of pN which is sufficient only for manipulating freely movable objects. In order to manipulate adsorbed nano-objects, forces of the order of tenths of nN are required [6].

Displacing the adsorbed nano-objects requires the use of an appropriately sized probe as it is provided, for instance, with a tip of an Atomic Force Microscope (AFM). AFM is the only method for directed relocation of sub-micron sized particles. Whereas it is suited for the manipulation of nanoscale assemblies of few particles, the approach is not suited for the simultaneous removal/rearrangement of a large amount of particles [7,8].

Several years ago, we proposed to use soft polymer films in order to induce motion of nano-particles adsorbed on top. For this, we constructed polymer surfaces that can change their shape and chemical composition during variation of external stimuli [9,10,11]. The particle simply follows the changing surface energy landscape. We demonstrated this process using certain types of polymer brushes composed of two different polymers. In this type of polymer films, the polymer chains are covalently attached with one end to a solid surface at high grafting density [12]. In the case when the film consists of two different polymers forming either mixed [13,14,15] (each of two polymers is attached with one end to a surface) or diblock-copolymer brushes (two polymers are connected in one chain that is attached to a surface with one of the ends) [16,17], the film can undergo nanophase separation with corresponding changes of surface topography and energy during variation of environmental conditions such as solvent quality, pH value, and temperature [18,19]. For generation of dynamic fluctuations in polymer brush topographies, the film is periodically exposed to solvents of different composition or temperature.

In order to change the polymer topography in air, i.e., when the polymer film is in a glassy state, one can make use of photo-isomerization reaction of azobenzene molecules integrated into polymer film. Azobenzene, consisting of two phenyl rings linked by an azo group (N=N), can undergo reversible photo-isomerization between a more stable *trans*- and a meta stable *cis*-conformation during illumination with light of an appropriate wavelength. This induces corresponding changes of physical properties of the molecules such as shape, free volume, and dipole moment [20]. Under irradiation with interference pattern, the film deforms following intensity or polarization distribution of the incoming light. This so-called surface relief grating formation (SRG) takes place just in air, at room temperature [21,22]. The mechanism behind this is most probably related to the local alignment of the azobenzene groups preferentially perpendicular to the electrical field vector through a rotational motion during multiple photo-isomerization cycles [23]. The formation of local phases of aligned azobenzenes coupled to the polymer matrix results in anisotropic photo-mechanical stress within a glassy polymer film large enough to induce macroscopic deformation of the polymer material [24,25,26,27]. In polymer brushes functionalized with azobenzene molecules, strong opto-mechanical stresses result in local opto-mechanical scission of the covalently bound chains [28,29,30]. This phenomenon can be used for an irreversible patterning of brushes on a nano-meter scale [31,32].

In order to reversibly switch the polymer topography, we have recently proposed a concept at which during irradiation with interference pattern the local distribution of the electrical field vector is changed by adjusting polarization of two interfering laser beams [33,34]. For this purpose, a home-made setup was used consisting of an optical part for the generation of different interference pattern coupled to an atomic force microscope (AFM) for simultaneous acquisition of the polymer topography response [35]. We have found that during changing of the local electric field distribution of the interference pattern, the polymer film topography varies resembling the motion of dunes in the desert.

Here we report on the motion of nano-particles adsorbed on top of azobenzene containing polymer film exposed to irradiation with different interference pattern. To inscribe topographical pattern, the polymer film was successively irradiated with interference pattern of PP (intensity interference pattern) and ±45° (polarization interference pattern) polarization, while simultaneously acquiring the topography change of the polymer film and the position of single adsorbed particles with AFM. The particles undergo either translational motion and move towards topography minima or rotational and are aligned at the bottom of the grating. We discuss experimental conditions required for particle displacement. The main parameter that influences particle/polymer interaction is the direction of irradiation. In cases when irradiation proceeds from “above”, i.e., from the side of polymer/particle interface, the particles partially sink into polymer surface and cannot be relocated any more due to strong adhesion. In contrast, when the sample is irradiated from “below”, i.e., through the glass substrate on which the polymer film is placed, the polymer particle interfacial energy is hardly modified due to strong adsorption of the incoming light within the polymer film.

## 2. Results and Discussion

**Figure 1:**
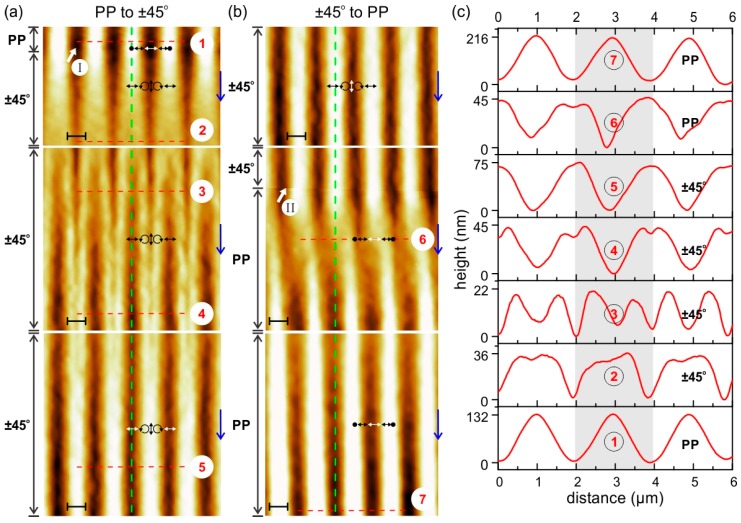
Switching of polymer topography using irradiation with PP to ±45° and PP interference pattern. (**a**) During scanning from top to bottom at the place marked by thick white arrow “I” the irradiation conditions were changed from PP to ±45° interference pattern; (**b**) At the position marked by thick white arrow “II” the interference pattern was changed back to PP polarization combination. The distribution of electrical field vector relative to the topography variation is shown by white arrows; (**c**) The grating profiles corresponding to the red dashed lines in (**a**,**b**).

The kinetic and morphology response of the Azo-PCMS polymer film on irradiation with different interference pattern was comprehensively investigated in our previous publication [33]. Here we show the characteristic behavior of the polymer topography on irradiation with different interference patterns (Figure 1).

As it can be seen in Figure 1, the topography maximum develops at the position where the local electrical field vector has vertical orientation in case of ±45° pattern (see white arrows in Figure 1) and intensity minima with PP pattern. The protocol of ascribing electrical field vector distribution to the topography variations is described elsewhere [34,35]. Upon exposure of the polymer films to irradiation with PP interference pattern and simultaneous acquisition of the topography development, the polarization was switched to ±45° interference pattern, the position of irradiation change is marked by a thick white arrow in Figure 1. The maximum possible height of the SRG inscribed in the film depends strongly on the nature of the interference pattern. It was found that inscribing with polarization interference pattern (±45°, RL) results in a maximal attainable SRG height of ca. 500 nm after one hour of irradiation (I = 100 mW/cm^2^, λ = 491 nm, polymer film thickness 500 nm, periodicity of IP is 2 μm). Irradiation with intensity interference pattern, e.g., with PP combination, induces a maximal SRG height of 100 nm. In this paper, we apply irradiation with smaller intensity (I = 30 mW/cm^2^). Together with the larger thickness of the polymer film, it results in slower SRG growth, so that the saturation in the topography change sets only after 15 h of irradiation. This is one of the important parameters needed for inducing motion of the adsorbed particles. We discuss this point together with the motion of the particles in more details below.

Summarizing this part, we state that the dynamic topographical changes within the polymer film can be induced by applying different combinations of interference pattern.

**Figure 2:**
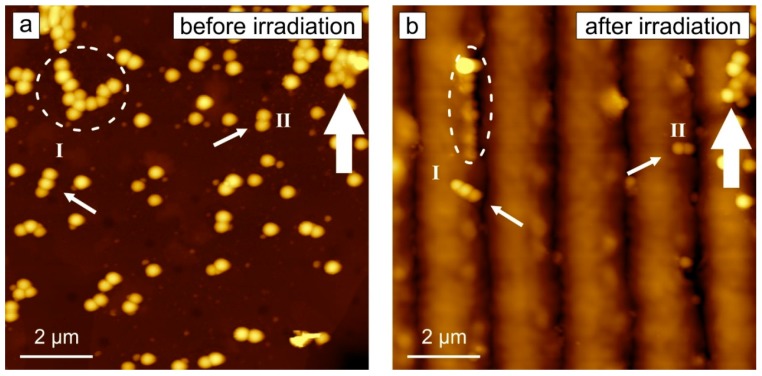
AFM micrographs of the polymer film covered with silica particles of 300 nm in diameter (**a**) before and (**b**) after successive irradiation with interference pattern of ±45° polarization combination (as described in Figure 1). White arrows indicate particles discussed in the text.

In cases when particles are adsorbed on top of such a dynamically fluctuating surface, the change in the position of the particles can be observed. In Figure 2, one can see the same area on the polymer film before and after irradiation with ±45° interference pattern. Silica particles of 300 nm in diameter deposited on the polymer film (see Section 3) form sparse coating (Figure 2a). Some of the particles can be found as single specimens, others form small aggregates. After irradiation, most of the particles are aligned along the topographical minima (Figure 2b). Some of the big clusters do not move (for example, see aggregate marked by bold white arrow), others rotate (see particles marked by white arrows I and II). The aggregate marked by dashed circle changes its shape from an “L” form before irradiation to an “I” after irradiation (Figure 2). The relocation of the particles was recorded ex situ, i.e., the position of the particles was measured before and after irradiation.

**Figure 3:**
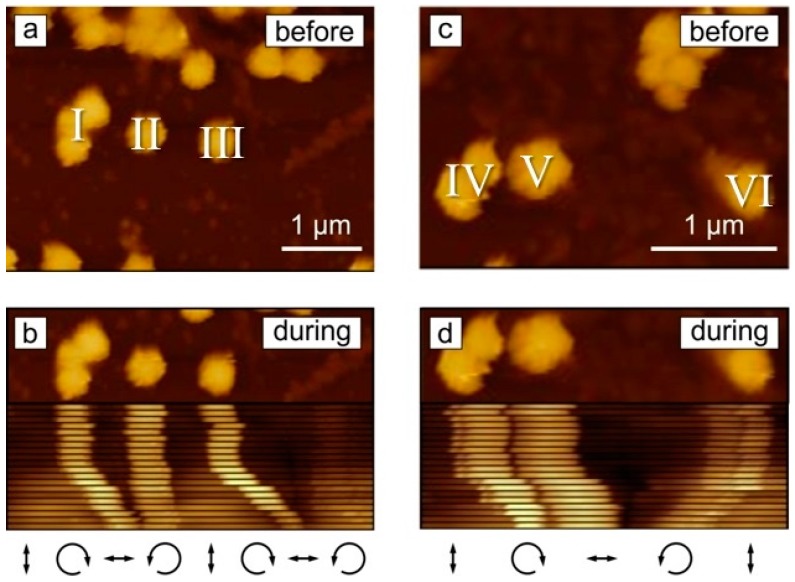
AFM micrographs of the particle displacement on a polymer film irradiated with interference pattern. The distribution of the electrical field vector relative to the particle position is shown below the micrographs. (**a**,**c**) Two selected areas before irradiation; (**b**,**d**) show corresponding areas during irradiation. The positions of the particles over irradiation time were cut out of the micrographs and immerged in one picture (**b** and **d**) to illustrate the particle displacement over time.

Figure 3 shows the displacement of some selected particles over time (the corresponding videos of the particle motion are presented in Supplementary Materials Videos S1 and S2). The particle aggregate marked by I in Figure 3a moves to the right towards the developing topography minima (Figure 3b), while the particle II, which is already close to the minima position, moves slightly to the left to get in to the topographical minima (Figure 3b). Particle III was moved towards the neighboring SRG minima (Figure 3b). Similar motion is observed for particles depicted as IV, V, and VI (Figure 3c,d). The local distribution of the electrical field vector together with the scheme of the topography variation is shown in Figure 3 below.

**Figure 4:**
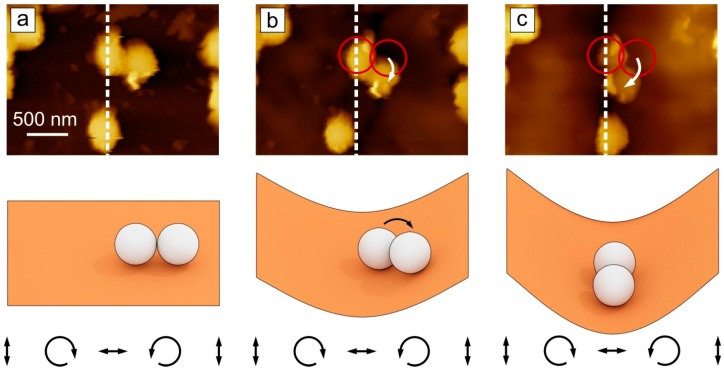
AFM micrographs and corresponding scheme (below) of the rotational displacement of two particles marked by red circles: (**a**) Before, (**b**,**c**) During irradiation. The distribution of the electrical field vector is depicted below. The dashed white line marks the position of the topography minima.

The rotational motion of two particles is shown in Figure 4 (corresponding movie is presented in Supplementary Materials Video S3). One of the two particles is adsorbed at the position where the topography minimum develops, the second particle rotates as shown by the white arrow in order finally to be positioned in the topographical minimum (Figure 4c).

**Figure 5:**
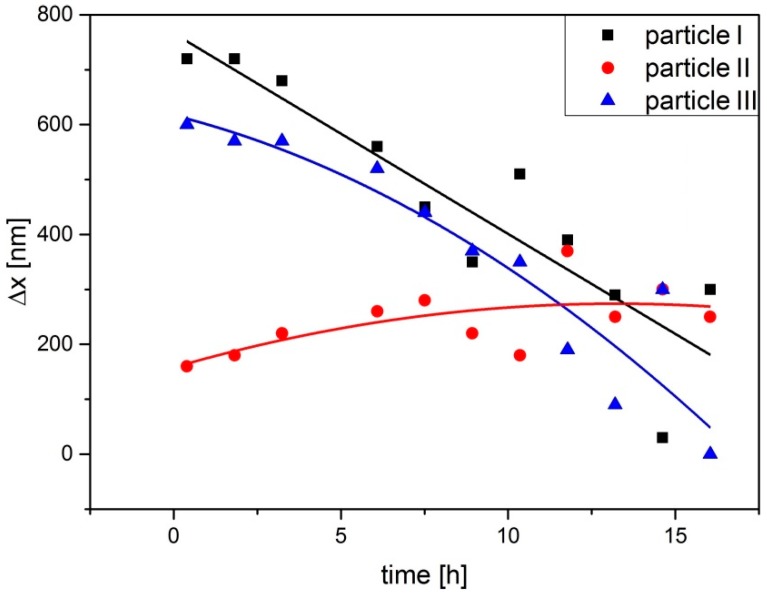
Displacement of the particles I, II, and III depicted in Figure 3a as a function of irradiation time. The displacement was measured relative to the topographical minima to which the particles move.

To analyze the velocity distribution of particles, the average displacement measured from the initial place relative to the position of the topographical minima was plotted as a function of time for several particles. Characteristic behavior is shown in Figure 5 for the particles I, II, and III from Figure 3b. The particles I and III move over ca. 650 nm during irradiation, while particle II—being adsorbed near the topography minimum—undergoes only moderate displacement.

The direction of the particle displacement (towards SRG minima) is opposite to the direction of the mass transport. Indeed, the topographical minimum develops when the polymer mass “flows” away from minima towards maxima, while the particles move towards minima. This behavior was also observed for the motion of flat objects on azobenzene containing polymer film irradiated by surface plasmons (SP) [36].

The SP near fields are generated within a metal pattern embedded in the polymer material. The intensity of the evanescent near field decays exponentially within the polymer film so that at the top most layer of the polymer topography, the intensity is almost vanished [37,38,39]. In our case, because of the low initial intensity and high extinction coefficient of the polymer, the intensity of the light at the polymer/air interface is quite low. Indeed, the intensity of the light as measured from the UV absorption spectrum of the dry polymer film adsorbed on a glass surface, drops by ca. 90% of the initial value. Therefore, we think that due to the low intensity at the polymer/air interface, the top most layer, i.e., “skin” of the polymer film, stays inactive and thus cannot take place in overall mass transport process in the polymer film. Instead the “skin” undergoes visco-elastic deformation in the opposite direction in order to compensate the opto-mechanical elastic stress. The objects adsorbed on the polymer film are then carried away of their initial position. If particles form clusters of the size larger than half of the SRG period, the aggregate could be moved since the mass transport is localized within this range. We should emphasize that the gravity cannot be taken into account as a driving force for the particle motion since the gravitational force on a silica particle of 300 nm in diameter is six orders of magnitude weaker than the adhesion force. In case when light shines through the polymer, the direction of the mass transport and the motion of the adsorbed objects are the same [11].

**Figure 6:**
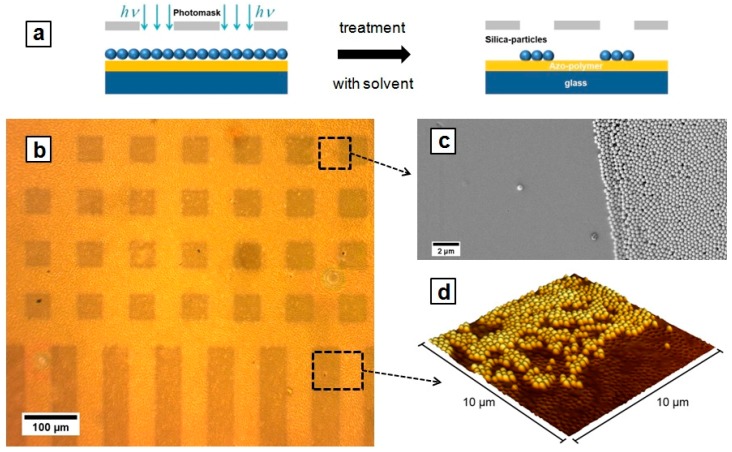
(**a**) Scheme of particle photo-immobilization. Silica particles adsorbed on a photosensitive polymer film are irradiated through a photosensitive mask followed by washing with ethanol. The particles exposed to irradiation stick to the polymer surface, while those adsorbed on non-irradiated area are removed during washing; (**b**) Optical micrograph of the polymer film patterned with silica particles; (**c** and **d**) SEM and AFM micrographs of the selected areas in (**b**), respectively.

We should also mention that in case of the irradiation from above that is from the side of the polymer/particle interface, the particles do not move. In this case, they sink partially within the polymer film in the areas of maximal intensity [40]. In this way, engulfed particles stick to the polymer surface so strong that they cannot be removed even during intensive washing, while the particles on the un-irradiated area are washed away [40]. In this way, one obtains a polymer surface structured with a particle pattern, the shape of which mimics the intensity distribution of irradiation. Figure 6 shows silica particles of 300 nm in diameter photo-immobilized on the azobenzene containing polymer film during irradiation through the photo-mask. The particles were adsorbed on the polymer film followed by irradiation through the mask (λ = 491 nm, I = 150 mW/cm^2^, t_irr_ = 3 h) and by washing with ethanol.

## 3. Materials and Methods

### 3.1. Photosensitive Materials

*Azo-PCMS*: Poly(chloromethylstyrene) was obtained using radical polymerization in solution (benzene) at 80 °C, by means of 2,2′-azoisobutyronitrile as an initiator (M_n_ ~ 5000 g/mol). Poly(chloromethylstyrene) was modified in a second step with 4-phenylazophenol [41]. The polymer substitution degree with azo-groups was higher than 99%, being calculated based on ^1^H-NMR spectrum (Supplementary Materials Figure S1).

**Figure 7:**
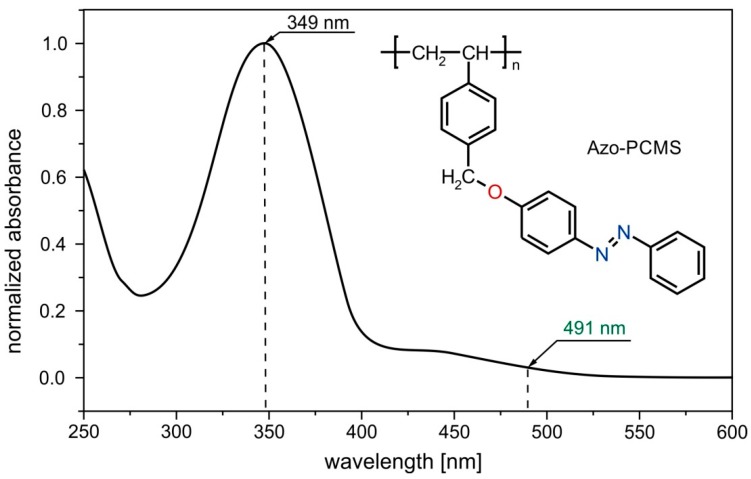
UV-Vis absorption spectrum of Azo-PCMS chloroform solution and chemical structure of Azo-PCMS. The irradiation wavelength of 491 nm is marked by a green arrow.

The glass transition temperature of the azo-polymer (Azo-PCMS, Figure 7) is T_g_ = 87 °C. The *trans-* state of the Azo-PCMS is characterized by the absorption band (π-π* transition) with a maximum at 349 nm while the *cis*-state has a characteristic adsorption peak situated at 442 nm (n-π* transition). The thermal relaxation of the *cis*-isomer (in dark) takes place within ca. 30 h.

The polymer film of 530 nm in thickness was prepared by spin coating of the 1,1,2-trichloroethane solution (*c* = 170 mg/mL). The polymer was irradiated with light of λ = 491 nm (I = 30 mW/cm^2^) applying interference pattern of different polarization and intensity. At this wavelength both *trans*- and *cis*-states adsorb, so that under illumination azobenzene undergoes multiple transitions and both types of isomers coexist.

### 3.2. Silica Particles

Silica particles of 300 nm in diameter were obtained from Sicastar (Partikeltechnologie GmbH, Rostock, Germany). The ethanol suspension of the particles was spin coated on a polymer film at 2000 rpm for 2 min.

### 3.3. In Situ AFM Connected Optical Setup

**Figure 8:**
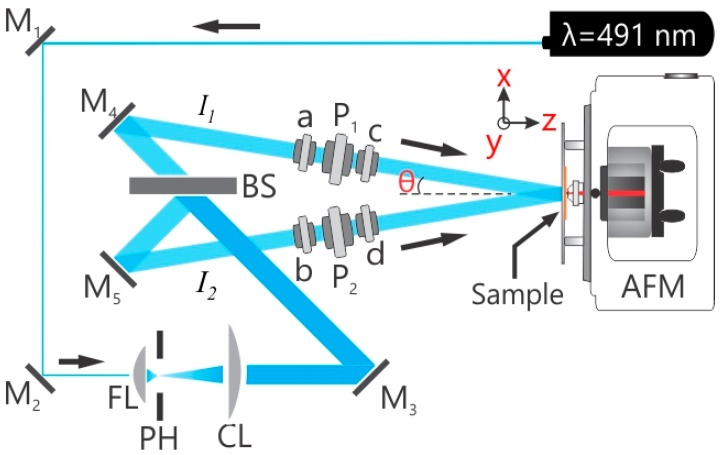
Experimental setups. In situ two-beam interferometric atomic force microscopy (IIAFM) used for inscribing the surface relief gratings (SRGs). FL: focusing lens; PH: pinhole; CL: collimating lens; (**a**,**b**)-half wave plates; (**c**,**d**)-either half wave or quarter wave plates and/or their combinations. P_1_, P_2_: Polarizers; AFM: atomic force microscope; BS: computer controlled beam shutter.

To achieve the photoinduced deformation in polymer films, a single beam and a two beam interferometer irradiation are used (Figure 8). The details of the set-up are published elsewhere [42]. In short, a continuous wave-diode pumped solid state laser (CW-DPSSL, 75 mW output power) with a wavelength of 491 nm is used (Cobalt Calypso™) for the two beam interference set-up. The holographic plane of 5 mm diameter central part is aligned at the polymer sample positioned within the AFM (PicoScan, Molecular Imaging, Phoenix, AZ, USA) for in situ measurements (Figure 8). The irradiation of polymer films is done from the glass side, while the polymer topography changes are recorded using an Atomic Force Microscope (AFM). All in situ AFM measurements were performed with 0.2 Hz scan speed, 512 scan lines per micrograph and each micrograph requires 43 min to complete. The polymer films were irradiated over 15 h using different polarization combinations.

### 3.4. Mask Irradiation

The photo-structuring of the silica particles was performed through an optical mask with squared holes and striped pattern of 50 μm in width. The irradiation was done using a laser of 491 nm wavelength (I = 150 mW/cm^2^) during 3 h. Afterwards, the sample was washed with ethanol in order to remove particles from the non-irradiated area.

## 4. Conclusions

We demonstrated the motion of adsorbed nano-particles induced by dynamic topography fluctuations. The particles were placed on top of the azobenzene containing polymer film and irradiated with light of λ = 491 nm wavelength. The irradiation was performed either through a mask or using different interference patterns. To inscribe dynamically changing topographical patterns, the polymer film was irradiated with an interference pattern of PP (intensity interference pattern) and ±45° (polarization interference pattern) polarization. The topography change of the polymer film and the positions of single adsorbed particles were recorded using the home-made set-up where the optical part is connected to the AFM. We have found that the particles move towards generated topography minima through either translational or rotational motion. The maximal displacement is at maximum a half of the SRG period. The particle motion is induced only when the sample is irradiated from “below”, i.e., through the glass substrate on which the polymer film is placed. In this case, the polymer particle interfacial energy is hardly modified due to strong absorption of the incoming light within the polymer film. In this way, the polymer surface undergoes little or no photo-isomerization and stays “inactive”. The particles move in the direction opposite to the mass transport, since they simply follow the direction of the deformation of the polymer “skin”. In contrast, when irradiation proceeds from “above”, i.e., from the side of polymer/particle interface, the particles partially sink into the polymer surface and cannot be relocated any more due to strong adhesion. They are photoimmobilized on the polymer surface.

## Supplementary Materials

Supplementary materials can be accessed at: http://www.mdpi.com/1420-3049/21/12/1663/s1.
