# Supplementary material for: Motion of Adsorbed Nano-Particles on Azobenzene Containing Polymer Films
Source: Molecules. 2016 Dec 3;21(12):1663. doi: 10.3390/molecules21121663 (PMC6274334; doi:10.3390/molecules21121663)
Supplement: Supplementary file 1 [file molecules-21-01663-s001.zip › molecules-154723-supplementary materials/molecules-154723-supplementary.pdf]

Molecules **2016**, *21*, 1663; doi:10.3390/molecules21121663

Supplementary Materials: Motion of Adsorbed Nano-Particles on Azobenzene Containing Polymer Films

Sarah Loebner, Joachim Jelken, Nataraja Sekhar Yadavalli, Elena Sava, Nicolae Hurduc and Svetlana Santer

The substitution degree of the chlorobenzyl groups from the starting PCMS is higher than 99%. In the ^{1}H-NMR spectrum of the Azo-PCMS the characteristic signal of the chlorobenzyl group (4.4 ppm) completely disappears, being replaced by a new signal (4.8 ppm) attributed to the sequence Ø-**CH**$_2$-O-Ø.

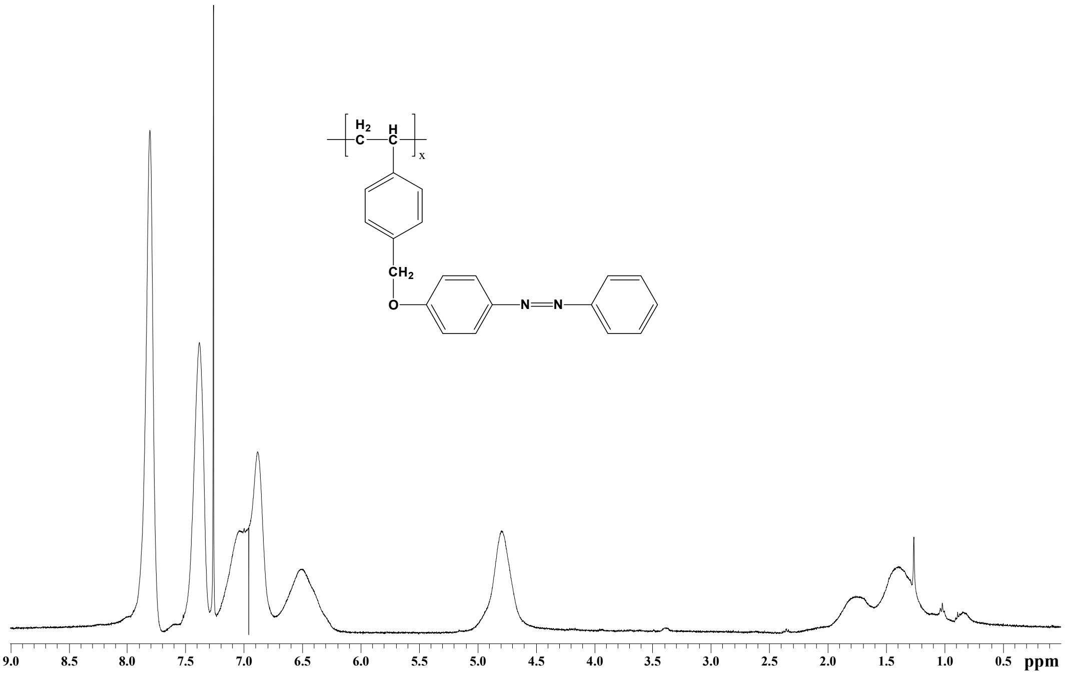

Figure S1. ^{1}H-NMR spectrum of the Azo-PCMS.
